# An Energy-Efficient Underground Localization System Based on Heterogeneous Wireless Networks

**DOI:** 10.3390/s150612358

**Published:** 2015-05-26

**Authors:** Yazhou Yuan, Cailian Chen, Xinping Guan, Qiuling Yang

**Affiliations:** 1 School of Electronic, Information and Electrical Engineering, Shanghai Jiaotong University, 800 Dongchuan RD. Minhang District, Shanghai 201100, China; E-Mails:yazhouyuan@sjtu.edu.cn (Y.Y.); xpguan@sjtu.edu.cn (X.G.); 2 Institute of Electrical Engineering, Yanshan University, 438 Hebei Street Haigang District, Qinhuangdao 066000, China; E-Mail: qiulingyang@hotmail.com (Q.Y.)

**Keywords:** heterogeneous wireless network, energy efficiency, adaptive packets scheduling, power control

## Abstract

A precision positioning system with energy efficiency is of great necessity for guaranteeing personnel safety in underground mines. The location information of the miners' should be transmitted to the control center timely and reliably; therefore, a heterogeneous network with the backbone based on high speed Industrial Ethernet is deployed. Since the mobile wireless nodes are working in an irregular tunnel, a specific wireless propagation model cannot fit all situations. In this paper, an underground localization system is designed to enable the adaptation to kinds of harsh tunnel environments, but also to reduce the energy consumption and thus prolong the lifetime of the network. Three key techniques are developed and implemented to improve the system performance, including a step counting algorithm with accelerometers, a power control algorithm and an adaptive packets scheduling scheme. The simulation study and experimental results show the effectiveness of the proposed algorithms and the implementation.

## 1. Introduction

Most recently, personnel safety has attracted more and more attention in the mining industry [1,2]. Underground mine accidents, which may cause serious personnel injuries, catastrophic mechanical failures and delays in production, bring about great financial losses for companies. In the case of coal mine accidents, a high-accuracy position system can greatly shorten the rescue time. Therefore, a low-power location system in an underground mine is urgently needed to improve the safety and productivity.

For localization in a coal mine, many techniques have been developed, including models of natural wave propagation in tunnels and different kinds of distance measurement methods [3,4]. However, the accuracy and low power consumption cannot be both satisfied. The nodes working in a tunnel will get reflected radiated energy from just about everything around the antenna. The reflected energy will constructively and destructively add or detract from the energy at any given point depending on the phase relationship at the point where the direct radiated signals and reflected signals sum together [5,6]. Meanwhile, technique research on the wireless propagation channels in tunnels with various kinds of practical models are also being carried out [7]. However, the mobile node must be battery-powered in a coal mine and has limited computing power. These models, due to the high complexity of the algorithm and the limited resolution and accuracy of the RF front-end, are not suitable for a coal mine. Therefore, one of the concerns is how to minimize the utilization of the power consumption of a mobile node with limited computational capability.

The received signal strength indicator (RSSI) is widely used in localization [8–12]. Whereas the signal is vulnerable to the surroundings, geological uncertainty and mobile obstructions are the primary sources of risk in underground tunnel construction. To some extent, it is hard to achieve accurate positing in real time in a coal mine by a practical network deployment.

In order to improve the localization accuracy, some solutions based on time-of-flight (TOF) are proposed to determine the distance between the transmitter and the receiver. However, the measurement requires at least three pieces of ranging information from the nodes [13]. How to effectively reduce the frequency of measurement and meet the requirements of energy efficiency are largely unexplored. On the other hand, the accelerometer is widely used in physical training and many other areas [14–16]. Considering the characteristics of a tunnel, the step counting algorithm could be used to estimate the walking distance of the miner. Additionally, the acceleration sensor is very suitable for an underground location system due to its low power consumption.

In this paper, a combined localization scheme with step counting and a TOF method based on a heterogeneous wireless network is designed to reduce the energy consumption of mobile nodes. The accuracy of the step counting algorithm can be improved by using the TOF locating technique to correct the cumulative error of step counting or to detect abnormal walking, such as turning back or stepping in the same place. Besides, a dynamically-adjusted transmitting power method for the mobile node is designed to reduce the power consumption further. Finally, an adaptive packets scheduling scheme according to the actual access demand with the time division multiple address (TDMA) algorithm is introduced.

The rest of the paper is organized as follows. Firstly, the system structure of the proposed location system is briefly described in Section 2. We proposed three algorithms in Section 3, including the step counting algorithm of accelerometer, the power control algorithm and the adaptive packets scheduling algorithm to achieve energy-efficient and accurate localization. In Section 4, the performance of the localization based on the proposed algorithms is analyzed. Finally, the conclusions are drawn in Section 5.

## 2. System Description and Problem Formulation

**Figure 1:**
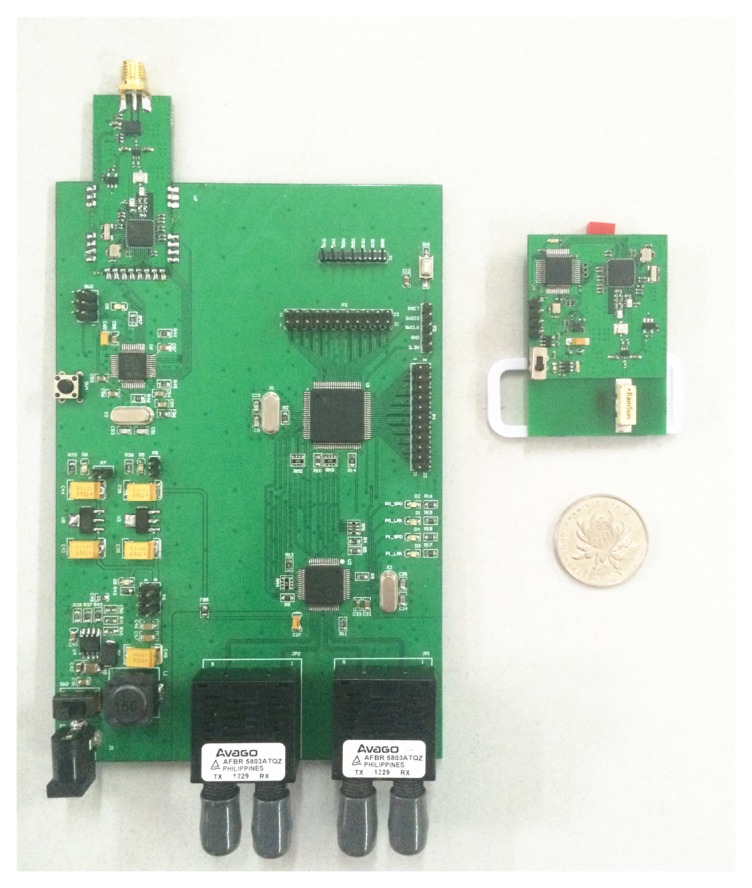
The prototypes of the BS and mobile positioning node (MN).

The network devices in an underground mine heterogeneous wireless network can be divided into three types, including the data processing center (DPC), base stations (BSs) and mobile positioning nodes (MNs). The DPC is a computer that is in charge of the historical location records and the tracking display. The BS consists of a STM32F207 processor for data processing, two fiber interfaces for wired data transmission and a wireless module, which includes a STM32L151 processor and a nanoLOCTRX Transceiver (NA5TR1) for wireless communication and TOF measure. NA5TR1 is a low-power, highly-integrated mixed signal chip with ranging capabilities utilizing the chirp spread spectrum (CSS); the supply current in transmit mode at 0-dBm output power is 30 mA and 2 µA when in low shut down mode; and the typical ranging accuracy is 2 m indoors [17]. The MN consists of a NA5TR1 transceiver, a STM32L151 processor and a MMA8652 sensor. The STM32L151 is a Cortex-M3-based ARM microcontroller. The architecture, combined with five low-power modes, is optimized to achieve extended battery life. When operating in run mode, the current is only 214 µA/MHz [18]. The MMA8652 is a low-noise, three-axis accelerometer, and the typical current is 27 µA when the output data rate (ODR) is 50 Hz. It contains a 32-sample first in first out (FIFO) buffer, which can save system power, by allowing the host processor to go into SLEEPmode, while the accelerometer independently stores the data [19]. Motion detection by MMA8652 is used to alert the STM32L151 that the MN is starting to work, and this feature is to ensure that MNs do not work without being warnedto save power. Figure 1 shows the prototypes of the BS (the left) and MN (the right). BSs collect the data of MNs via air interface and exchange the information with other BSs and with the data processing center via fiber optic Ethernet. MNs are responsible for calculating the miner's proceeding steps and uploading these step data to the BSs. All of the wired equipment synchronizes the network time through the IEEE 1588 protocol to hold the reference time that will be used by the packets scheduling scheme.

**Figure 2:**
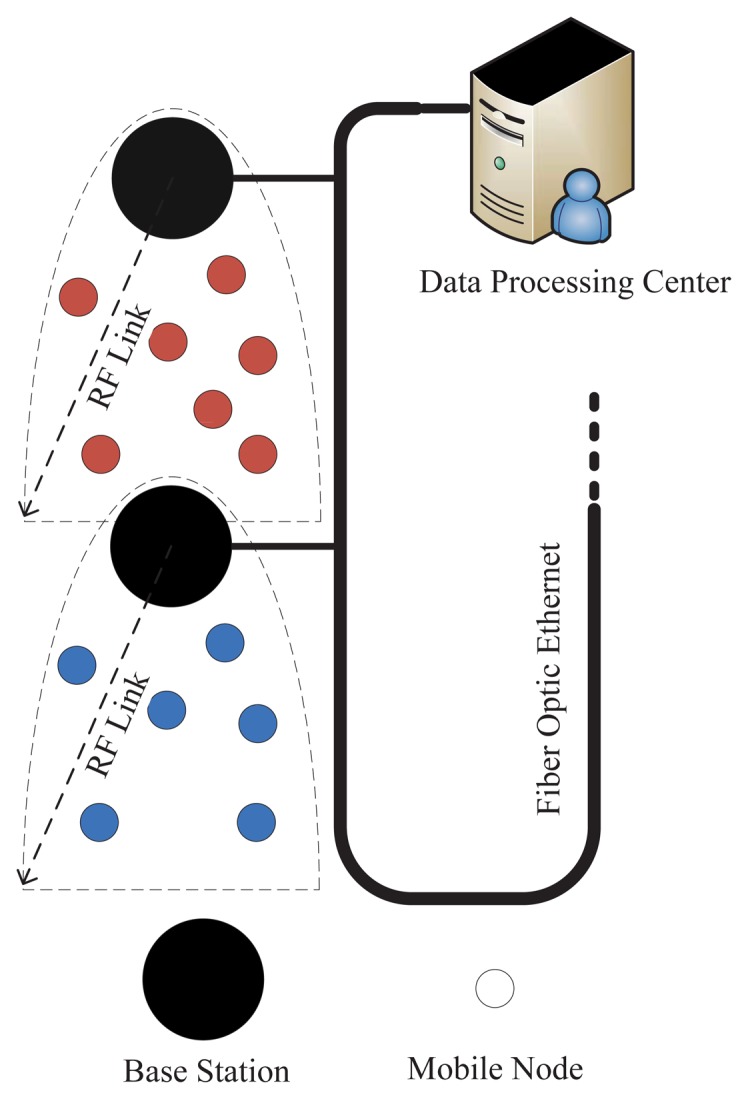
System architecture.

Directional antennas are used by the BSs, which could reduce the complexity of the localization algorithm, because the MN just needs to measure the distance from only the connecting BS, whose location is already known, to find its own location. Moreover, to avoid an interfering signal with the same frequency among BSs, different communication frequencies between adjacent BSs are adopted repetitively, which could improve the spatial reuse of the radio spectrum and affect the capacity of the BSs. BSs determine the travel direction of the MN by historic record information and estimate whether the next period needs to change the communication channel according to the latest location of the MN. The block diagram of the overall system is illustrated in Figure 2. There is no need to consider the power consumption of the BSs, because they are powered by a power line. Therefore, the BSs can transmit radio frequency signals with maximum power to achieve maximum transmission distance.

## 3. Key Algorithms and System Design

### 3.1. Step Counting Algorithms for Accelerometers

Normally, the distance walked by the miner is obtained simply by monitoring the radio frequency signals in the existing positioning systems. However, the accuracy and low-power consumption cannot be both satisfactory. In order to save power consumption and obtain relatively precise location information, in this section, a combination of step counting and a wireless communication method with the accelerometer is proposed.

The STM32L processor gathers acceleration data by FIFO interrupt and processes the data by the Kalman filter algorithm after a fixed data buffer, that is, most of the time, STM32L remains in sleep mode to save power. After that, a proper threshold is set to obtain the walked steps of the miners. The NA5TR1 transceiver remains in shut down mode until it is time to upload the step data via air interface to the BS.

The BSs calculate the walking distance of the miners by the relationship between steps and the single step distance, then the position information is estimated by comparing the node address information with historical data.

### 3.2. Power Control Algorithm

**Figure 3:**
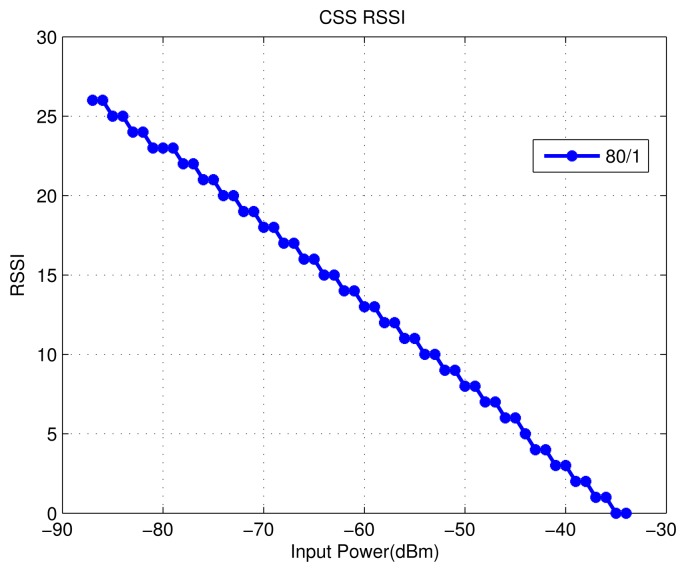
Chirp spread spectrum (CSS) received signal strength indicator (RSSI) vs. input power.

The transmit power level at the air interface is separately adjustable for MNs, and the lower power level allows MNs to save energy and extend battery lifetime. The power range of NA5TR1 is adjustable from zero to 63, which is equivalent to actual transmitting power. The chip also provides an RSSI value, which is a relative measure of the received signal power. The relationship between RSSI and the input power is shown in Figure 3.

It is concluded that the RSSI values assume a linear relationship with the input power; therefore, the received power can be estimated by the RSSI values. Therefore, how to obtain a more accurate RSSI value becomes a problem that must be considered.

**Figure 4:**
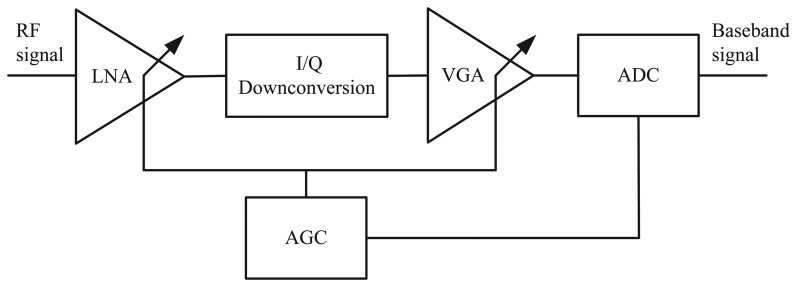
Block diagram of the nanoLOC receiver chain with automatic gain control (AGC).

Figure 4 is the block diagram of the RF front-end NA5TR1. The automatic gain control (AGC) and RSSI are directly linked together, as the gain set by the AGC is interpreted as the RSSI value. The lower the measured power, the higher the AGC gain. However, a high AGC value means that the background noise is also amplified. As is well known, the less the influence of noise on the ADC, the greater the accuracy of the sampling value. Therefore, it is favorable to get a larger SNR value in the terminal. SNR is widely used in the wireless environment and usually is referred to as a power ration between a signal and background noise, which can be described as:

(1) $$\text{SNR}=\frac{P_{R}}{P_{N}}=\frac{P_{T}g}{P_{N}}$$

with:

$$g=\frac{G_{T}G_{R}\lambda^{2}}{{\left(4\pi \right)}^{2}d^{2}}$$

where *G_T_* is the gain of the transmitting antenna; *G_R_* is the gain of the receiving antenna; λ is the wavelength; and *d* is the propagation distance. Therefore:

(2) $$\text{SNR}=\frac{P_{T}G_{T}G_{R}\lambda^{2}}{{\left(4\pi \right)}^{2}d^{2}P_{N}}$$

With a certain antenna gain and communication frequency in the communication system, *SNR*is proportional to *P_T_* when the distance and noise is determined:

(3) $$\text{SNR}\propto \frac{P_{T}}{d^{2}P_{N}}$$

A relatively accurate path loss can be obtained through monitoring the signal strength of the beacon frame sent by BSs, since the BSs always transmit radio signals at maximum power according to the aforementioned analysis.

In an interactive communication between a BS and an MN at one time, the effect of noise and the path of RF transmission are supposed to be unchanged due to the time in communication being so short and the relatively position being fixed between two devices. The path loss is supposed to be a constant, which will be proven in Section 4.2.

After obtaining the path loss (*P_L_*) value, the minimum transmit power (*P_T_*) can be set as:

(4) $$P_{T}=P_{L}+S$$

where *S* is the receiver sensitivity and set to −95 dBm at *BER* = 0.001 [17].

In order to have a certain margin in the system, *P_T_* rounds up to a reasonable integer.

### 3.3. Adaptive Packets Scheduling Scheme

Considering the characteristics of the localization algorithm, the tunnels are divided into walking areas and fixed working regions. In the walking areas, the method of location combines step identification along with TOF; while in the fixed working regions, the step counting algorithm is stopped to reduce the cumulative error, and the frequency of TOF is lessened to cut down on power consumption.

Due to the variable numbers of MNs within the BS's communication range, the time of the MN accessing the media is needed to adjust dynamically. This paper proposes an adaptive TDMA scheduling scheme according to the actual access demand. The time scale for transmission is divided into multiple variable time slots (TSs) to make maximum use of the spectrum. Each TS provides variable time determined by the packet length and offset time for accessing the media to transmit one packet. The basic unit of measurement for times slots is one time slot unit (TSU), which is defined as the time it takes to transmit one octets, and one TSU takes 32 µs when the data rate is defined at 250 kbit/s.

To administrate, allocate and dedicate TSs, the BSs provide TS information to MNs within their communication coverage range. Each MN has to communicate with the first BS to register for access information by the carrier sense multiple access (CSMA) algorithm before entering the tunnel. The BS arranges communication with the TS in accordance with the FAFA (first access first arrange) method. After joining the heterogeneous network, the MN communicates with the BS according to the given TDMA TS until getting another arrangement frame.

#### 3.3.1. Combined Localization Scheme

**Figure 5:**
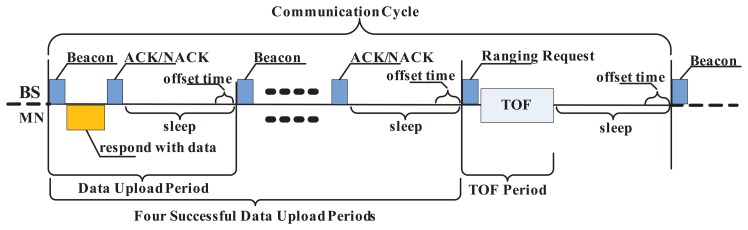
Timing diagram in the communication cycle.

At the location stage, the BSs send beacon messages to the MNs within the reachable range and determines which node should make a response according to the scheduled time sets. The MN with the address matched is waiting for the beacon to synchronize the data transfer. When receiving the beacon frame, it sends a message that contains the step data payload back to the BS. The defect of the localization method based on the acceleration sensor is the cumulative error, hence it is necessary to calibrate the step counting result regularly by the TOF method. For each registered MN, the communication cycle is divided into five periods, as shown in Figure 5, which consist of four data upload periods (DUPs), along with one TOF period (TOF-P). Each period contains a sleep time, which is composed of a fixed sleep time along with a field of offset time.

It is noticed that while in the sleep time, the processor is still working periodically to gather the acceleration data. The basic unit of the individual period is TSU, and the time of the period taken is variable due to the offset time decided by the actual access demand. After four successful DUPs, the BS sends a data packet with a ranging request to the MN to be located. Then, a symmetrical double-sided two-way ranging (SDS-TWR) methodology is implemented to obtain a reliable position [17]. The TOF-P is used mainly to calibrate the cumulative error of an irregular situation. If the communication failure scenarios appear in TOF-P, the BS calculates the MN's location by historic records until another calibration process period to update the location history.

#### 3.3.2. Communication Frame

To guarantee the portability of embedded program code, all frames are designed based on the Medium Access Control (MAC) data frame, and the frame formats are defined by the application payload field of the MAC frame. However, ranging measurement packets formatted with TOF are not included here,because they are fixed and must apply to the ranging methodology of the nanoLOC chip. The fields of the designed general frame appear in a fixed order, which shall be formatted as illustrated in Tables 1, 2, 3 and 4. It is worth noting that the source address field, communication channel field, offset time field and step data field may not be included in all frames.

The description of notations in the Tables are as follows: FRAMTYP, frame type field, indicates that a packet type being a beacon frame, ACK frame, NACK frame or DATA frame; SRCADDR, source address field, indicates which node sends the packet; DESTADDR, destination address field, indicates which node the packet is sent to; COMCHAN, communication channel field, indicates that the wireless channel will be used when the next communicating period; OFFTIME, offset time field based on TSU, this field is an indicator of the amount of time besides the fixed sleep period that must elapse until the current transmission is complete. It consists of two octets, which could represent up to 2097.12 ms; STEPDATA, step data field, indicates that the accumulation of step data since last step counting reset.

#### 3.3.3. Time Slot Allocated Algorithm

The medium access time is dynamically adjusted by the BSs. Each BS allocates access to the TS according to the frame records of the connecting MNs within its communication range, also by monitoring communication records shared by the adjacent BSs. Since the length of the various communication frames is fixed, the transmission time is respectively fixed. Each BS holds a scheduled time set *P^m^* defined as Equation (5), which is used to denote the set of the time already assigned.

(5) $$\begin{array}{cc} p^{m}=\overset{r}{\underset{j=1}{\text{∑}}}P_{j}^{m}, & n=1,2,\ldots ,r \end{array}$$

where the superscript *m* is the ID of the BS (the *m_th_* BS can be expressed as *BS^m^*); $P_{j}^{m}$ is the scheduled time subset, which is defined as Equation (6); and subscript *j* is the *j_th_* subset corresponding to the addresses of MNs within *BS^m^*'s communication range.

(6) $$P_{j}^{m}=\left\{T_{j}^{m}+T_{f},T_{j}^{m}+T_{f}+T_{p}^{l}+T_{o}^{j}\right\}$$

where

*T_f_* is the fixed sleep period;
$T_{o}^{j}$ is the offset time; *j* stands for the *j_th_* scheduled time;
$T_{p}^{l}$ is the packet transmitting time, separately determined by the packet length *l*;
$T_{j}^{m}$ is the start access time of the corresponding MN; *j* stands for the *j_th_* MN's access time.

The BSs reallocates the TS of the MN under the two scenarios.

Scenario 1, after receiving the step data sent by the MN:
  Assuming that the *i_th_* MN is communicating with the *BS^m^*; when receiving the data, the *BS^m^* updates the *P^m^* by removing the elapsing TS, which can be expressed as $p^{m}=p^{m}-p_{i}^{m}$, records the end time $T_{i}^{m}+T_{f}+T_{p}^{l}+T_{o}^{i}$ as $T_{i^{\text{end}}}^{m}$ and creates a new subset $P_{i^{\text{new}}}^{m}$ to denote the expected time subset. The whole process is summarized in Algorithm 1.

**Algorithm 1:** After receiving the step data sent by the MN.
1.**Start:**2.**Let**
$P_{i^{\text{new}}}^{m}=\left\{T_{i^{\text{end}}}^{m}+T_{f},T_{i^{\text{end}}}^{m}+T_{f}+T_{p}^{l}\right\}$3.**If**
$P_{i^{\text{new}}}^{m}\cap P^{m}=0$4. Goto Label 15.**Else**6.**Update**
$P_{i^{\text{new}}}^{m}=\left\{T_{i^{\text{new}}}^{m}+T_{f},T_{i^{\text{new}}}^{m}+T_{f}+T_{p}^{l}+T_{o}^{i^{\text{new}}}\right\}$7. Adjust 
$T_{o}^{i^{\text{new}}}$ by TSU, to satisfy 
$P_{i^{\text{new}}}^{m}\cap P^{m}=0$8.**Label 1:**9.**Let**
$P_{i}^{m}=P_{i^{\text{new}}}^{m}$10. 
$P^{m}=P^{m}\cup P_{i}^{m}$11.**End**
Scenario 2, a new MN wants to connect with the BS:
  When there is a new MN to join the *BS^m^*, the BS records the start access time and other necessary information shared by the former *BS^m^*^−1^, creating a new time subset $P_{i^{\text{new}}}^{m-1}$ to denote the time subset, removing the offset time allocated by the former. The whole process is summarized in Algorithm 2.
  When *P^m^* is determined, the BS will calculate and allocate TSs according to the up-to-date *P^m^*.

**Algorithm 2:** A new MN wants to connect with the BS.
1.**Start:**2.**Let**
$P_{i^{\text{new}}}^{m-1}=\left\{T_{i^{\text{former}}}^{m-1}+T_{f},T_{i^{\text{former}}}^{m-1}+T_{f}+T_{p}^{l}\right\}$3. For the network time is synchronized4. 
$T_{i^{\text{new}}}^{m}=T_{j^{\text{former}}}^{m-1}$5.**Update**
$P_{i^{\text{new}}}^{m-1}=\left\{T_{i^{\text{new}}}^{m}+T_{f},T_{i^{\text{new}}}^{m}+T_{f}+T_{p}^{l}\right\}$6.**If**
$P_{i^{\text{new}}}^{m-1}\cap P^{m}=\emptyset$7. Goto Label 18.**Else**9.**Update**
$T_{i^{\text{new}}}^{m-1}=\left\{T_{i^{\text{new}}}^{m}+T_{f},T_{i^{\text{new}}}^{m}+T_{f}+T_{p}^{l}+T_{o}^{i^{\text{new}}}\right\}$10. Adjust 
$T_{o}^{i^{\text{new}}}$ by TSU, to satisfy 
$P_{i^{\text{new}}}^{m-1}\cap P^{m}=\emptyset$11.**Label 1:**12.**Let**
$P_{i^{\text{new}}}^{m}=P_{i^{\text{new}}}^{m-1}$13. 
$P^{m}=P^{m}\cup P_{i^{\text{new}}}^{m}$14.**End**

#### 3.3.4. Scheduled Time Reliability Assurance Scheme

A transmitted frame does not always reach its intended destination due to the imperfect nature of the radio medium. Different data transmission situations are illustrated as follows:

- Successful data transmission:
  The successful data transfer can be described as four processes.
  (1) The BS transmits the beacon frame to the MN via the air interface. When waiting for the data packet sent by the MN, the BS starts a timer that will expire after the data wait duration based on the TSU, which is called *T_DWD_* and defined by the application sub-layer.
  (2) After receiving the beacon frame, the MN deliveries the beacon frame to the next higher layer to get the RSSI value by which the path loss can be calculated. Then, the MN sends the step data back to the BS and starts a timer that will expire after the ACK wait duration based on the TSU, which is called *T_AWD_* and defined by the application sub-layer.
  (3) The BS application sub-layer receives the step data from the MN before its timer expires and then disables and resets the timer *T_DWD_*. Hereto, the data transfer is completed, and the BS issues an ACK frame with scheduling information to the MN.
  (4) After the MN receives the ACK frame, it will disable and reset the timer *T_AWD_*, record the communication schedule given by the BS and clear the step data; then, it shall sleep and wake according to the schedule until getting another ACK frame for rearrangement.
- Lost step data frame:
  However, the data transfer may fail when the timer *T_DWD_* of the BS expires before the step data are received, and the BS will not receive the step data frame. In this situation, the BS responds with a NACKframe with schedule information to the MN; whereafter, the MN receives the NACK frame from the BS before its timer expires and then disables and resets the timer *T_AWD_*, whereafter it records the communication schedule given by the BS. In this case, the MN will retain the step data till the next successful communication, and it shall sleep and wake according to the schedule until getting another ACK/NACK frame for rearrangement.
- Lost ACK/NACK frame:
  Another situation exists in transmission, which is the acknowledgment frame is lost. Since the BS will send an ACK/NACK frame back to the MN whether it receives the step data frame or not, another issue that cannot be ignored is that the acknowledgment frame is lost in the data transfer process. If the MN does not receive the ACK/NACK frame before its timer *T_AWD_* expires, the MN would sleep and wake according to the latest schedule record until getting another ACK/NACK frame for rearrangement. In this case, the MN will retain the step data until the next successful communication.

## 4. Implementation and Experiment Results

### 4.1. Kalman Filter for Step Counting

The MNs are fixed on the underground miners' outer ankles, so that we can capture the maximum acceleration, which is easy to obtain. Normally, the acceleration is of three dimensions, that is horizontal direction (labeled as *x*-axis), vertical direction (labeled as *y*-axis) and lateral direction (labeled as z-axis). Here, the lateral acceleration is not considered due to the small effect on the count.

**Figure 6:**
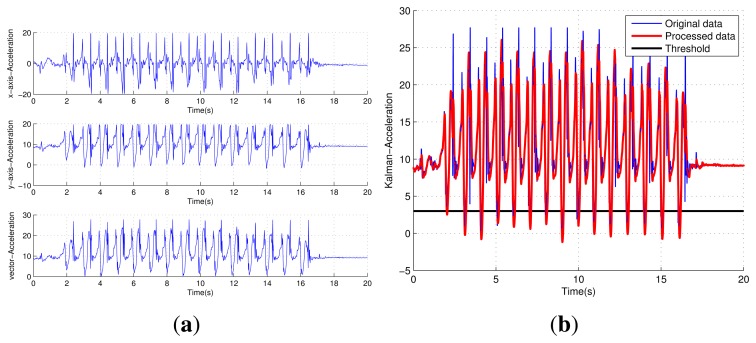
(**a**) The raw acceleration in the measurement; (**b**) the filtered acceleration data.

The graph as shown in Figure 6a represents the raw acceleration obtained from the data collection terminal. Due to the uncertainty of the walking state, there is a lot of noise existing in the raw data. It is hard to get accurate step counts. Therefore, it is necessary to take some measures to process the data. The Kalman filter is an efficient recursive filter for real-time processing and a suitable method to this special situation [20,21]. As shown in Figure 6b, the acceleration vector data are smoothed after the Kalman filter. Meanwhile, the noise disturbance is mostly removed and the accuracy of determining steps gets better improvement than before.

### 4.2. P_L_ Constant

The experiment was carried out in a corridor environment. Node A sent a packet to Node B with a certain transmit power. Node B recorded the RSSI value of the received packet. Meanwhile, Node B sent a packet back at the same transmit power to Node A as a response. Node A recorded the received RSSI value in the same way. This interactive experiment was conducted every three meters as Node B moving away from Node A, while the first datum was sampled at one meter from Node A.

**Figure 7:**
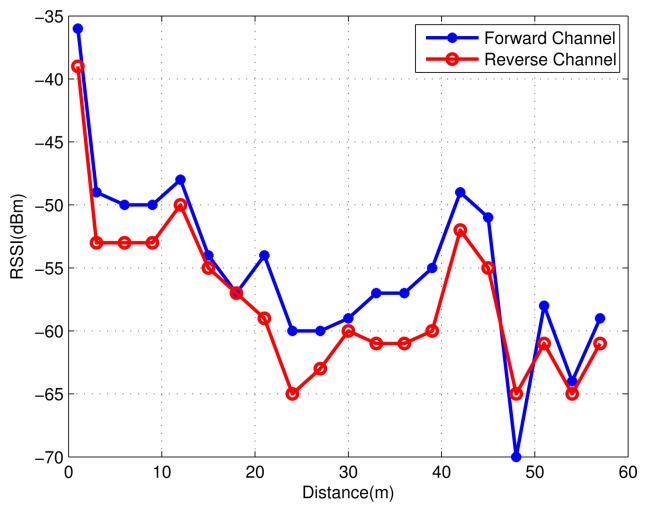
The value of the RSSI record by two nodes.

As shown in Figure 7, the packet transmitted from Node A to Node B is defined as the forward channel and Node B to Node A as the reverse channel. Due to the differences among devices, the antenna and PCB, the values of the RSSI in the forward channel and reverse channel are almost at the same level within a certain error range. This conclusion is also suitable for the value of the received power, since the estimate of the received power is from the RSSI in the nanoLOC chip.

Path loss (*P_L_*) is defined as:

(7) $$P_{L}=P_{T}-P_{R}$$

where *P_T_* is transmitting power and *P_R_* is receiving power. It is assumed that *P_T_* and *P_R_* are consistent during one interactive communication; the value of *P_L_* is then considered to be a constant.

### 4.3. Analysis of Power Consumption

In this section, we evaluate the system performance with different parameter values and analyze the simulation results to find a proper value for the combined location method.

Considering that the lengths of packets defined in this paper are similar, the energy taken by transmitting or receiving a packet, denoted as *P_unit_*, is expressed as a standard unit of energy consumption for the purposes of analysis. Meanwhile, as the acceleration sensor has a very low power consumption and the processing time of STM32L is very short, the effects of them can be ignored.

SDS-TWR is used in the nanoLOCTRX Transceiver for high precision positioning, and it takes five interactions between the BS and MN. Moreover, the BS needs to send an ACK/NACK frame with a time slot arrangement to the MN after the SDS-TWR process in order to inform the MN of the next communication time. Therefore, the TOF-P process requires a total of six packets interacting, while in DUP, it is three times, as mentioned in Section 3.3.4. Then, we can have:

(8) $$P_{\text{TOF}}=6P_{\text{unit}}$$

(9) $$P_{\text{DPU}}=3P_{\text{unit}}$$

#### 4.3.1. Impact of the Proportion of DUPs over TOF-Ps on Power Consumption

One hundred communication cycles are taken for analysis in this section, including *n* DUPs and *m* TOF-Ps. The power consumption of total communication cycles can be shown as:

(10) $$P_{\text{total}}=mP_{\text{TOF}}+nP_{\text{DUP}}=3\left(2m+n\right)P_{\text{unit}}$$

We denote the proportion of DUPs and TOF-Ps as *k*. And *m* and *n* have the following relationship:

(11) n/m=k,k∈{1,2,…,20}

(12) m+n=100

**Figure 8:**
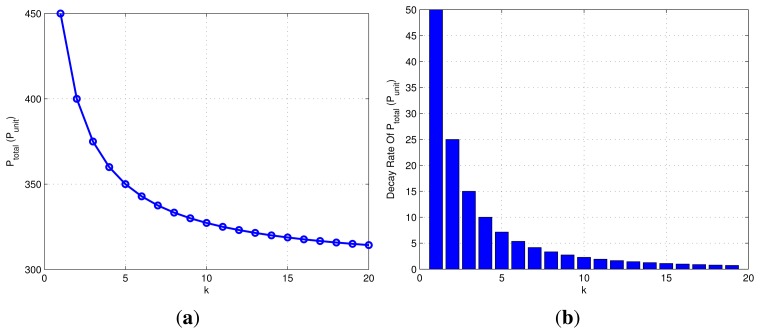
(**a**) Power consumption of the 100 communication cycles; (**b**) power decay rate of different values of *k*.

The *P_total_* under different *k* is represented in Figure 8a. It can be seen that the overall power consumption decreased gradually as the *k* increases. In the combined localization algorithm, the higher the value of *k*, the greater the cumulative error and response time of the system (see further analysis below). Therefore, the maximum *k* here is set to 20.

Figure 8b shows the corresponding power decay rate of different *k*. When *k* is less than four, the *P_total_* changes rapidly. With the increase of *k*, the change rate of power is getting smaller. That is, the effect of reducing power consumption is not obvious.

Remark 1: In this paper, The *P_total_* varies slowly when the decay rate of *P_total_* is smaller than eight.

#### 4.3.2. Impact of Fixed Sleep Period on Power Consumption

**Figure 9:**
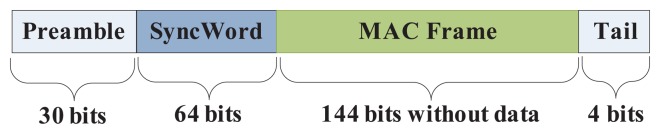
Schematic view of the physical layer protocol data unit (PPDU).

According to the configuration of NA5TR1, each physical layer protocol data unit (PPDU) consists of four frame fields: a preamble, a sync word, a MAC frame and a tail [22], as illustrated in Figure 9. In order to simplify the analysis, we take the typical data packet as a reference. Assuming that all of the frames have the same length, and each packet contains five bytes of data, the total length of each frame is (30 + 64 + 144 + 5 × 8 + 4) bits, that is 35.25 octets. Therefore, the time taken by transmitting or receiving a packet via air interface is *T_trans_* = 35.25 × *TSU*(32 *us*) = 1.128 ms.

**Figure 10:**
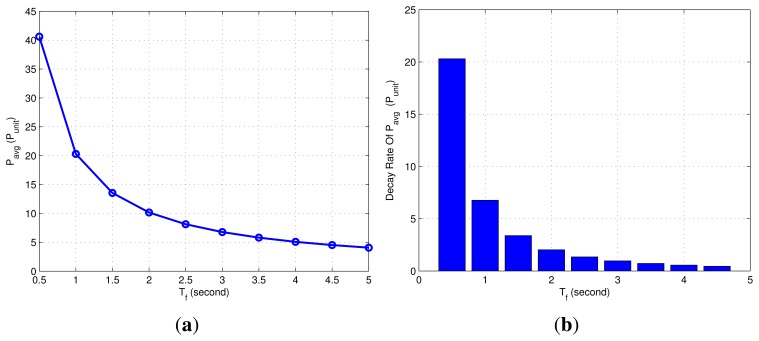
(**a**) The average cycle power consumption based on different sleep times; (**b**) the average cycle power attenuation rate based on different sleep times.

Since the fixed sleep period is *T_f_*, the average power consumption of TOF-P is expressed as *T_trans_* × 6*P_unit_*/(*T_f_* + *T_trans_* × 6)( *where T_f_* ≫ *T_trans_*) ≈ 1.128 × 6*P_unit_*/*T_f_*, and the average power consumption of DUP is about 1.128 × 3*P_unit_*/*T_f_*. The number of DUPs is set to four by the previous analysis; then the average cycle power dissipation is *P_avg_* = (1.128 × 3 × 4 + 1.128 × 6)*P_unit_*/*T_f_* = 20.304*P_unit_*/*T*_f_. We take 0.5 s as the increment to analyze the effect of different sleep times on the overall power consumption in a communication cycle, which is given in Figure 10a. It is shown from Figure 10a that the average cycle power consumption is on the decline with the increase of fixed sleep period.

The corresponding average cycle power attenuation rate is given in Figure 10b. It is shown from Figure 10b that when *T_f_* is less than two, the power consumption changes rapidly. Additionally, with the increase of time for the fixed sleep period, the average cycle power attenuation rate is not obvious.

Remark 2: In this paper, *P_avg_* varies slowly when the decay rate of *P_avg_* is smaller than two.

#### 4.3.3. Error Bound under Normal Communication Conditions

**Figure 11:**
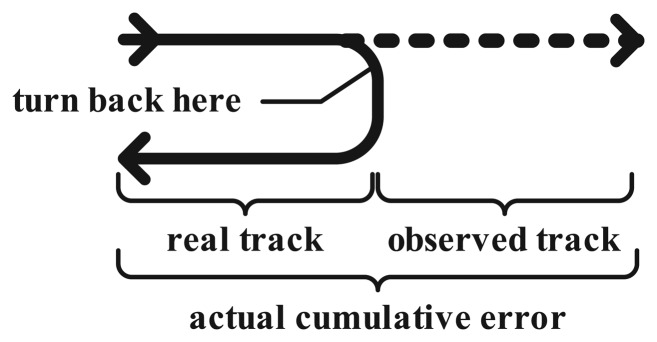
The scenario with the maximum error.

The maximum error happens when the miner wearing the MN changes direction immediately after TOF-P. Because the location method is based on step counting in the subsequent *N* periods, the system still estimates the position of the miner by historical record; therefore, it is unable to determine the real position until the next TOF-P. The maximum error that occurs in this scenario is shown in Figure 11.

Since *T_f_* ≫ *T_trans_*, the maximum error can be expressed as:

(13) $$E_{\text{max}}=2vT_{f}N$$

where *v* is the walk speed of miners; *T_f_* is the fixed sleep period, and *N* is the number of DUPs. Taking 4 and 1 m/s for the initial value of *N* and *v*, respectively, *E_max_* gets a result of 8*T_f_* m.

**Figure 12:**
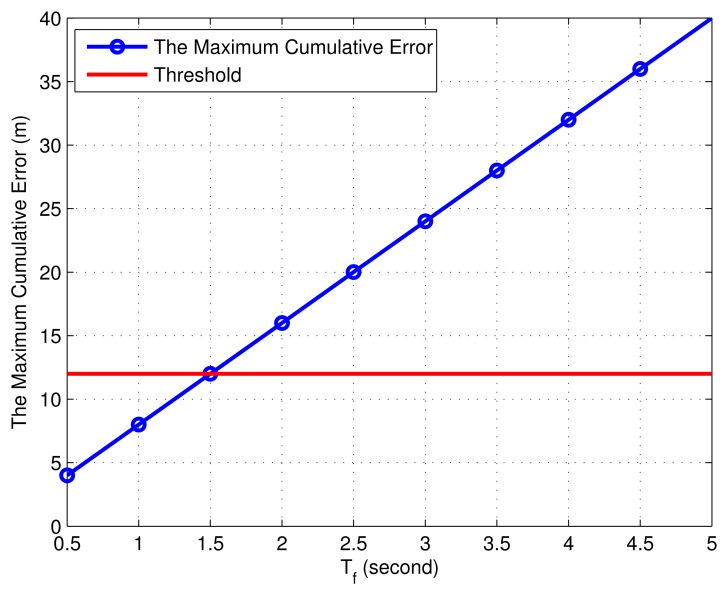
The impact of the fixed sleep period on the maximum error.

We take 0.5 s as an increment value to analyze the different times of the fixed sleep period on the impact of maximum error, which is given in Figure 12. It is shown from Figure 12 that the maximum cumulative error increases linearly with the increase of *T_f_*. According to the actuality in the project, the threshold of the maximum cumulative error is set as 12, and the *T_f_* value is 1.5 at this time.

To evaluate the system in an actual situation, a test was carried out along a 40-m corridor. Two participants (the average stride lengths are respectively 0.6 m and 0.64 m) were asked to walk forward and turn round at a certain period. The test consists of 27 periods with the *T_f_* set to 1.5 s. A status LED on the MNs flashed to indicate the data uploading process and TOF process during each period. To obtain the actual distance from the BS, another participant walked along with the one who wore the sensor node and made marks on the floor when the status LED flashed. Then, we used the tape to measure the distance and recorded the data.

**Figure 13:**
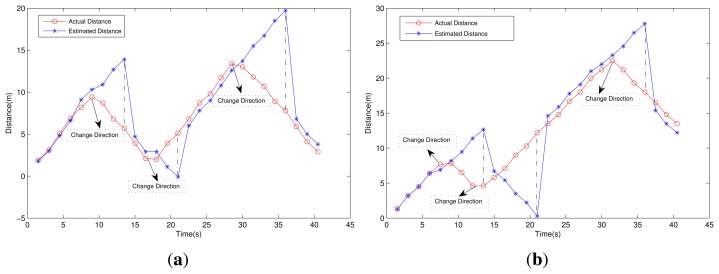
The relationship between distance and time: (**a**) Participant 1; (**b**) Participant 2.

As shown in Figure 13, we can see that in the normal condition, the cumulative error is very small and can be adjusted during TOF-P. The maximum cumulative error occurs in the cycle when the MN changes direction immediately after the TOF-P. In a real-world situation, the movement takes a while when the participant turns round, so the step counts during the period are less than the normal walking condition, which leads to smaller cumulative errors. The TOF process may introduce errors into the observations and affect the measurement accuracy more or less in TOF-P, but the errors are minor and not the decisive factors for maximum cumulative error. It is noteworthy that in most cases, the miners walk straightly along the tunnel or stay in the working region, so the cumulative errors are very small in most situations.

In conclusion, a longer fixed sleep period and a greater number of DUPs will save more battery energy, but they will also bring in bigger system cumulative error and longer response time. Four DUPs along with one TOF and *T_f_* = 1.5 s is a trade off for the performance of the combined location system; the maximum error is 12 m in this situation, but the accuracy is still higher than the traditional localization method based on RSSI under the same network deployment. Furthermore, its reliability is also improved accordingly.

#### 4.3.4. Comparison of the Estimated Power Consumption in Different Location Methods

**Figure 14:**
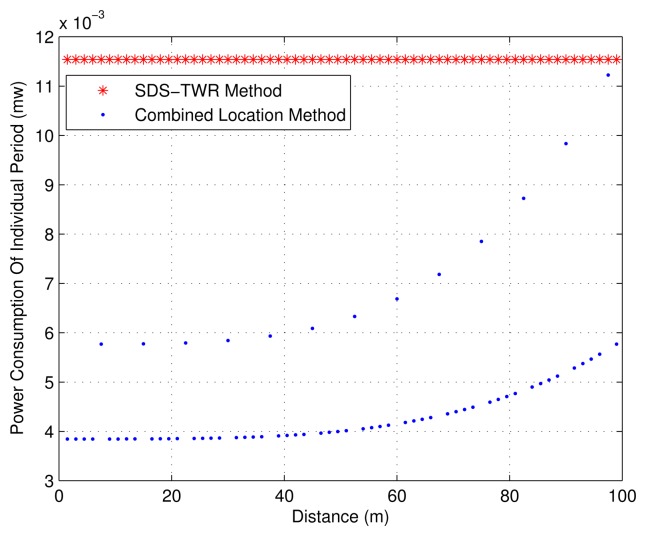
The estimated power consumption of different location methods.

According to the previous analysis, the communication cycle is divided into four DUPs along with one TOF-P, and *T_f_* is set to 1.5 s. To facilitate the analysis, we use the model of [23] to estimate the minimum transmitting power by the power control method, and the instantaneous power is denoted by *P_i_* (*i* ∈ N*). Assuming that MN moves away from *BS^m^* at the speed of 1 m/s and normal transmitting power is used when it is 99 m from *BS^m^*, we set a packet transmitted at this power as the standard unit of energy consumption, expressed as $P_{\text{unit}}^{'}$. Since the power consumption in receive mode is always almost the same as the transmitting power consumption in normal mode, it is also defined as $P_{\text{unit}}^{'}$ and unchanged in any case. During DUPs, the power consumption should be expressed as $P_{i}+2P_{\text{unit}}^{'}$ (send one packet and receive two packets), while during TOF-Ps, it is $3P_{i}+3P_{\text{unit}}^{'}$ (send three packets and receive three packets). For the location method based on SDS-TWR, the corresponding period's power consumption is denoted as $P_{\text{TOF}}=6P_{\text{unit}}^{'}$. The comparison of the power consumption in the SDS-TWR method based on TOF and the combined location method with power control is given in Figure 14. It is shown from Figure 14 that the combined location method can reduce the power consumption to a great extent.

## 5. Conclusions

This paper has presented an energy-efficient underground localization system by combining an acceleration sensor for step counting and wireless communication technology. A corresponding adaptive packets scheduling scheme based on a heterogeneous wireless network is also proposed to make the MNs access the media dynamically according to the actual access demand. Additionally, a practical power control method is implemented by the MNs to reduce the power consumption further. Since the proposed localization algorithm does not rely on the propagation characteristics of the wireless signal, it can adapt to different tunnel environments without rebuilding the wireless signal transmission model. The simulation results proved that the correct choice of the proportion of DUPs and TOF-Ps or a proper value for the time of the fixed sleep period could save much battery energy and obtain accurate location information.

## Figures and Tables

**Table 1:** General frame format. TSU, time slot unit.

**FRAMTYP**	**SRCADDR**	**DESTADDR**	**COMCHAN**	**OFFTIME**	**STEPDATA**
Octets: 1	0/2	2	0/1	0/2	0/1

**Table 2:** ACK/NACK with data frame format.

**FRAMTYP**	**DESTADDR**	**COMCHAN**	**OFFTIME**
Octets: 1	2	1	2

**Table 3:** Data frame format.

**FRAMTYP**	**SRCADDR**	**DESTADDR**	**STEPDATA**
Octets: 1	2	2	1

**Table 4:** Beacon frame format.

**FRAMTYP**	**SRCADDR**	**DESTADDR**
Octets: 1	2	2

## References

[1] Pang Q., Lu Y. Design application on ERP system of coal mine safety.

[2] Zhang Y., Yang W., Han D., Kim Y.I. (2014). An integrated environment monitoring system for underground coal mines-wireless Sensor Network subsystem with multi-parameter monitoring. Sensors.

[3] Dudley D.G., Lienard M., Mahmoud S.F., Degauque P. (2007). Wireless propagation in tunnels. IEEE Antennas Propag. Mag..

[4] Castiblanco J.A., Seetharamdoo D., Berbineau M., Ney M., Gallée F. Surface Boundary conditions for lossy dielectrics to model electromagnetic wave propagation in tunnels.

[5] Emslie A., Lagace R., Strong P. (1975). Theory of the propagation of UHF radio waves in coal mine tunnels. IEEE Trans. Antennas Propag..

[6] Ndoh M., Delisle G.Y. Underground mines wireless propagation modeling.

[7] Dudley D.G. (2005). Wireless propagation in circular tunnels. IEEE Trans. Antennas Propag..

[8] Patwari N., Wilson J. (2010). Rf sensor networks for device-free localization: Measurements, models, and algorithms. IEEE Proc..

[9] Wilson J., Patwari N. (2012). A fade-level skew-laplace signal strength model for device-free localization with wireless networks. IEEE Trans. Mob. Comput..

[10] Tang J., Chen Y., Chen L., Liu J., Hyyppä J., Kukko A., Kaartinen H., Hyyppä H., Chen R. (2015). Fast Fingerprint Database Maintenance for Indoor Positioning Based on UGV SLAM. Sensors.

[11] Wong W., Liew L.S., Lai C.H., Liu L. (2013). Accurate Indoor Positioning Technique Using RSSI Assisted Inertial Measurement. Future Information Communication Technology and Applications.

[12] Wang J., Gao Q., Yu Y., Cheng P., Wu L., Wang H. (2013). Robust Device-Free Wireless Localization Based on Differential RSS Measurements. IEEE Trans. Ind. Electron..

[13] Rohrig C., Muller M. Localization of sensor nodes in a wireless sensor network using the nanoLOC TRX transceiver. In.

[14] Amini N., Sarrafzadeh M., Vahdatpour A., Xu W. (2011). Accelerometer-based on-body sensor localization for health and medical monitoring applications. Pervasive Mob. Comput..

[15] Taraldsen K., Chastin S.F., Riphagen I.I., Vereijken B., Helbostad J.L. (2012). Physical activity monitoring by use of accelerometer-based body-worn sensors in older adults: A systematic literature review of current knowledge and applications. Maturitas.

[16] Sun B., Wang Y., Banda J. (2014). Gait Characteristic Analysis and Identification Based on the iPhone's Accelerometer and Gyrometer. Sensors.

[17] Nanotron Technologies GmbH (2009). NanoLOC TRX Transceiver (NA5TR1) Datasheet. NA-09-0230-0388-2.3.

[b18-sensors-15-12358] 18.Ultra-low-power 32-bit MCU ARM-based Cortex-M3, 128 KB Flash, 16 KB SRAM, 4 KB EEPROM, LCD, USB, ADC, DAC. Datasheet—Production Data, DocID17659 Rev 9, November 2013 Available online: http://www.st.com/web/catalog/mmc/FM141/SC1169/SS1295/LN962/PF259988 (accessed on 14 May 2015).

[b19-sensors-15-12358] 19.Xtrinsic MMA8652FC 3-Axis, 12-Bit Digital Accelerometer. Data Sheet: Technical Data Rev. 2.0, February 2013 Available online: http://www.freescale.com/webapp/sps/site/prod_summary.jsp?code=MMA8652FC (accessed on 14 May 2015).

[20] Tao W., Liu T., Zheng R., Feng H. (2012). Gait analysis using wearable sensors. Sensors.

[21] Zhao N. (2010). Full-featured pedometer design realized with 3-Axis digital accelerometer. Analog Dialogue.

[22] Nanotron Technologies GmbH (2008). NanoLOC TRX Transceiver (NA5TR1) User Guide. NA-06-0230-0385-2.00.

[23] Rappaport T.S. (1996). Wireless Communications: Principles and Practice.

